# Evaluating Undergraduate Learning in an Ophthalmology Rotation: A Study Using Virtual Questionnaires

**DOI:** 10.7759/cureus.72965

**Published:** 2024-11-04

**Authors:** Adriano C Faneli, Dillan C Amaral, Laura Cheidde, Luanna Guimarães A Gonzales, Rodrigo A Torres, Murilo B Souza, Ricardo D. C Oliveira

**Affiliations:** 1 Medicine, BAHIANA - School of Medicine and Public Health, Salvador, BRA; 2 Medicine, Universidade Federal do Rio de Janeiro, Rio de Janeiro, BRA; 3 Medicine, City University of São Paulo (UNICID), São Paulo, BRA; 4 Medicine, Salvador University, Salvador, BRA; 5 Ophthalmology, HOBrasil, Salvador, BRA; 6 Ophthalmology, Federal University of Bahia, Salvador, BRA

**Keywords:** clinical competence, competency-based education, medical students, ophthalmology, teaching

## Abstract

Background

Medical school ophthalmology education is often insufficient for non-ophthalmologist physicians to achieve proficiency in managing ophthalmic cases.

Objective

This study evaluates learning during a clinical rotation in a Brazilian medical school that implemented a two-week ophthalmology rotation.

Methods

This quantitative and qualitative study involved all eligible students for the rotation. Two different questionnaires were administered before and after to assess knowledge, understanding, and problem-solving abilities in ophthalmology. Each questionnaire contained 11 theme-paired questions developed according to the International Council of Ophthalmology curriculum and guidelines for medical student education. The primary qualitative outcome was a 30% improvement in scores between the pre- and post-rotation tests, as defined by two ophthalmology professors who developed the questionnaire. Data was analyzed anonymously using STATA BE 18 software, and questionnaire reliability was measured using Kuder-Richardson Formula.

Results

Of the 69 eligible students, 36 (52.17%) completed both tests. The internal consistency of the pre-and post-rotation questionnaires was acceptable, with Kuder-Richardson Formula scores of 0.66 and 0.64, respectively. Although scores significantly improved from 6.97 pre-rotation to 7.72 post-rotation (p=0.01), the mean score only improved by 10.76%, falling short of the 30% target. Scores improved significantly in the Red Eye and Primary Care Attention from 0.49 to 0.78 (p<0.01), but decreased in Anatomy and Physiology of the Posterior Segment (from 0.80 to 0.67, p<0.01) and the Anatomy and Physiology of Extraocular Muscles (from 0.92 to 0.79, p=0.04). No statistically significant difference was observed in the Anatomy and Physiology of the Anterior Chamber.

Conclusions

This study concludes that undergraduate students learning during an ophthalmology rotation was unsatisfactory. However, this is an exploratory study; further research with a larger sample size is necessary.

## Introduction

Preparing medical students to provide ophthalmological care has been a challenge in medical education, with a documented decrease in instruction in this area over the decades [1-4]. In 2014, a study revealed that only 18% of medical schools had a compulsory internship in a clinical ophthalmology rotation, representing a significant decline of 68% compared to 2000 [5]. Several factors have been identified as responsible for this negative trend, including increasingly overloaded curricula, greater competition among medical specialties for teaching time, lack of support from ophthalmology educators, and the reluctance of curriculum committees to recognize the utility of a mandatory ophthalmology rotation [6-9]. Consequently, medical graduates are ill-prepared to handle their patients' ophthalmological complaints.

The International Council of Ophthalmology (ICO) proposes a competency-based approach to medical education that emphasizes knowledge of ocular diseases, anatomy, and ocular manifestations of systemic diseases, as well as the essential clinical skills to assess visual acuity, visual fields, extraocular movement, and other indicators of ocular health [10]. However, despite these guidelines, many medical students still experience discomfort regarding their level of theoretical and practical ophthalmological knowledge [11-14]. This lack of exposure to ophthalmology may lead some students to dismiss the possibility of pursuing this specialty as a career. Furthermore, physicians who graduate from medical school without adequate experience in ophthalmology may not fully grasp the importance of ocular health and the breadth of ophthalmological practice as generalist doctors [15].

Although the need for formal ophthalmology training in medical schools is evident, studies utilizing a qualitative and a quantitative outcome of the learning in ophthalmology are lacking. Given the gap in ophthalmological educational literature, our work is exploratory research that aims to evaluate ophthalmology learning during a rotation at a medical school in Brazil. This study's primary objective is to assess medical students' learning outcomes during a two-week ophthalmology rotation. Specifically, we sought to evaluate the effectiveness of this rotation in enhancing students' knowledge of ophthalmology using a pre- and post-test design. We hypothesized that students would significantly improve their knowledge following the rotation. This approach is grounded in the competency-based education framework proposed by the ICO, which emphasizes acquiring theoretical knowledge and developing essential clinical skills necessary for diagnosing and managing common ophthalmic conditions. The pre-and post-tests were designed based on the ICO guidelines to capture key ophthalmology competencies, including foundational knowledge and problem-solving abilities relevant to clinical practice.

## Materials and methods

Population

For this cross-sectional and quantitative study, fourth-year medical students were chosen, who participated in an ophthalmology rotation consisting of standardized theoretical lectures and practical outpatient clinical activities lasting two weeks with a workload of 23 hours between June 2022 and December 2022 at an ophthalmological hospital in Brazil. None of the students had any prior formal exposure to ophthalmology. Sixty-nine students were eligible to respond to the questionnaire, but only 36 answered the pre- and post-rotation tests, constituting the sample for the statistical analysis. The students received the pre-rotation questionnaire 15 days before the initial date of the rotation. The post-rotation questionnaire was sent to the students one day after the end of their rotation.

To reduce the risk of bias, the participants received the questionnaire via institutional and personal e-mail and could not access their colleagues' answers. Only students who answered the pre- and post-rotation questionnaires were included, so there is no missing data since students who answered only one of the questionnaires were excluded from the analysis. Participants who responded to the pre-rotation questionnaire after the start of the rotation were excluded. There was no limit on the days to answer the post-rotation questionnaire.

The study was approved by the Institutional Review Board of Bahiana School of Medicine (No. 5,768,569) and performed in accordance with the ethical standards outlined in the 1964 Declaration of Helsinki and its later amendments or comparable ethical standards. All participants provided informed consent willingly and with full awareness. This article was previously posted to the Research Square preprint server on December 19, 2023.

Questionnaire development, validation, and outcomes

The development of the pre- and post-rotation questionnaires was grounded in the ICO guidelines for undergraduate ophthalmology education [10]. These guidelines emphasize the acquisition of core competencies in both theoretical knowledge and practical skills necessary for managing common ophthalmic conditions. Based on these principles, the questionnaires were designed to assess students' understanding across several key domains, including Anatomy and Physiology of the Posterior Segment, Red Eye and Primary Care Attention, Anatomy and Physiology of the Anterior Chamber, and Anatomy and Physiology of Extraocular Muscles.

The initial pilot test consisted of 40 questions. After analyzing the pilot results, 22 questions were selected for the final version of the pre- and post-tests. This refinement process ensured that the questions chosen aligned with ICO competencies provided adequate differentiation between various levels of student knowledge, and eliminated any ambiguous or irrelevant items. Both content validity and expert validation were achieved through repeated reviews by two experienced ophthalmologists with over a decade of teaching experience. These experts also ensured that the questions reflected the educational objectives of the ophthalmology rotation.

To assess learning, the final version of the questionnaires, consisting of 11 questions, was administered before and after the rotation. Each questionnaire had a maximum score of 11 points. The study's primary outcome was defined as a 30% improvement in the mean score from the pre- to post-rotation questionnaires. This threshold was established a priori by the same two ophthalmologists based on the expected learning gains for students with no prior formal exposure to ophthalmology. The difficulty level of the questions was set to challenge the students while remaining achievable within the two-week rotation.

All data were analyzed anonymously, and the results were aggregated, preserving the students' identities.

Statistical analysis

Stata BE 18 software (StataCorp LLC, College Station, TX, USA) was used for statistical analysis. The Shapiro-Wilk test was used to test the normality of the data. Kuder-Richardson Formula was used to measure the reliability and internal consistency of the two questionnaires. The paired t-test was used to compare the mean scores of the pre-rotation and post-rotation questionnaires and their scores across the four domains.

## Results

Sixty-nine individuals were eligible to participate in this study, but only 44 underwent the pre-rotation test. Eight of these did not complete the post-rotation test after concluding their rotation. Therefore, 36 participants answered both questionnaires, representing 52.17% of the eligible population. The internal consistency of the pre-rotation questionnaire was 0.66 and 0.64 for the post-rotation questionnaire. Table 1 illustrates participants' pre- and post-rotation scores and descriptive and comparative statistics. 

**Table 1:** Descriptive Statistics and Paired T-test Results Comparing Pre-Rotation and Post-Rotation Scores.

Statistics	Pre-Rotation Scores	Post-Rotation Scores
Mean	6.97	7.72
Standard Deviation	2.13	2.13
Mode	5	7, 8
Paired T-test (p-value)	0.037	

Two participants achieved the maximum score. One of them obtained the total score in both the pre- and post-rotation tests, whereas the other reached it only in the post-rotation test. Although the overall 30% score improvement was not achieved, 12 students attained a 30% improvement in their scores. The average score improvement was 17.2% for the entire sample.

Table 2 presents the overall mean and domain scores for pre- and post-rotation questionnaires. There was a significant increase in the overall mean score, from 6.97 in the pre-rotation questionnaire to 7.72 in the post-rotation questionnaire (p<0.01). Students showed a statistically significant improvement in the Red Eye and Primary Care Attention domain, with scores increasing from 0.49 pre-rotation to 0.78 post-rotation (p<0.01). The performance in the Anatomy and Physiology of the Posterior Segment decreased significantly, from 0.80 pre-rotation to 0.67 post-rotation (p<0.01). Similarly, Extraocular Muscles' Anatomy and Physiology scores also reduced, from 0.92 pre-rotation to 0.79 post-rotation (p=0.04). There was no statistically significant difference in the scores for the Anatomy and Physiology of the Anterior Chamber domain, with pre-rotation scores of 0.42 and post-rotation scores of 0.56.

**Table 2:** Domain-Specific Scores for Pre- and Post-Rotation Questionnaires

Domain	Number of Questions		Mean Score by Student		
	Pre-Rotation	Post-Rotation	Pre-Rotation	Post-Rotation	p-value *
Anatomy and Physiology of the Posterior Segment	4	4	0.80	0.67	p<0.01
Red Eye and Primary Care Attention	4	3	0.49	0.78	p<0.01
Anatomy and Physiology of the Anterior Chamber	1	2	0.42	0.56	p=0.10
Anatomy and Physiology of Extraocular Muscles	1	2	0.92	0.79	p=0.04
*Paired t-test					

## Discussion

In this study, we developed and applied two questionnaires to measure the learning efficacy of an ophthalmology rotation in a Brazilian medical school. To the best of our knowledge, this is the first study to assess the effectiveness of learning in an ophthalmology rotation through pre- and post-rotation questionnaires. Students showed a 10.76% improvement from the pre-rotation to the post-rotation score. Although the mean improvement in score from 6.97 to 7.72 (p<0.01) was statistically significant, the qualitative outcome of at least a 30% improvement was not achieved. Sixty-nine students received the first questionnaire, 44 responded to the pre-rotation questionnaire, and only 36 completed both questionnaires. The pre-rotation questionnaire had an internal consistency of 0.66, and the post-rotation questionnaire had an internal consistency of 0.64, indicating acceptable reliability. Despite the overall score increase after the rotation, a significant increase was observed only in the Red Eye and Primary Care Attention domain. Furthermore, there was a statistically significant decrease in the Anatomy and Physiology of the Posterior Segment scores and the Anatomy and Physiology of Extraocular Muscles domains, demonstrating poor learning in these areas. Although there was a numerical increase in the score of the Anatomy and Physiology of the Anterior Chamber domain, it was not statistically significant.

Ophthalmic education is relevant for most clinical subspecialties. Ophthalmology proficiency enables physicians to diagnose, treat, and refer, whenever necessary, common ophthalmic pathologies. Moreover, ophthalmological examination provides clinically valuable insights into other highly prevalent systemic diseases that affect microvasculature circulation, such as diabetes and hypertension [1]. A study reports that 80% of family medicine residents describe their capacity for assessing and managing common ophthalmic conditions as “somewhat comfortable” or “not at all comfortable" [16]. Another study shows that despite the availability of a distance vision chart and an ophthalmoscope, only 44% of United Kingdom primary care physicians felt comfortable utilizing the ophthalmoscope, and 39% were using the visual distance chart, not according to the manufacturer's instructions [17]. In our study, the increase of score in the post-rotation questionnaire was 17.2%, below the 30% increment expected to consider the learning of the rotation as sufficient, demonstrating that learning during ophthalmology rotation is insufficient. Prior studies have reported that medical doctors are uncomfortable with ophthalmological management. Still, they did not use an actual metric to measure and quantify learning during the ophthalmology rotation. Our study is the first objective measurement of the inadequacy of ophthalmological learning during medical training. Due to this insufficiency, non-ophthalmologist physicians have difficulty in managing ophthalmological cases. This translates into increased healthcare costs and delays in referring time-sensitive ophthalmological diseases, such as retinoblastoma, age-related macular degeneration, and retinal detachment [18,19].

Previous studies have shown that students are more comfortable performing primary care ophthalmological exam tasks, such as measuring visual acuity, assessing pupillary reflexes, and conducting visual field examinations [20]. However, they face difficulties with more advanced procedures like fundoscopy, slit-lamp examinations, and intraocular pressure assessment [20]. An analysis of Canadian medical students found that 83.3% and 90.7% of undergraduates rated their assessment of visual acuity and pupillary reflexes, respectively, as competent [21]. In contrast, only 52.3%, 44.8%, and 19.9% of students considered themselves competent in fundoscopy, slit-lamp examination, and intraocular pressure assessment, respectively [21]. In another study, 96.1% of medical interns could confidently diagnose conjunctivitis, 57.4% could not diagnose strabismus, and 79.8% could not diagnose macular degeneration [20]. Our study demonstrates these limitations, as the only domain with a statistically significant increase in score was Red Eye and Primary Care Attention, indicating that learning in the current ophthalmological rotations has been effective only in teaching these specific domains of ophthalmological conditions, such as conjunctivitis, measuring visual acuity, and pupillary reflexes. Nevertheless, students had poor scores after the rotation in the Posterior Segment and Extraocular Muscle diseases, showing that there is a need for improvement in ophthalmology education regarding diseases that affect the posterior segment, such as diabetic retinopathy, and clinically significant examination procedures for these domains, such as fundoscopy.

This work has some limitations. Firstly, our study had a small sample size: students from only one medical school in Brazil. However, this study is an exploratory experiment, and further work should aim for a larger sample size. One of the primary limitations is the relatively low response rate, with only 52.17% of eligible students completing both the pre- and post-rotation questionnaires. This limited participation may introduce bias, as those who responded might represent a group more engaged or interested in ophthalmology.

Furthermore, the small sample size reduces the generalizability of our findings, limiting the conclusions that can be drawn regarding the overall effectiveness of the ophthalmology rotation. Future studies should aim to mitigate this issue by increasing the sample size through multi-institutional collaborations, thus allowing for a more diverse and representative cohort. Additionally, we did not measure students' perceptions of the rotation and their learning or collect data on what suggestions students would make to enhance learning during their ophthalmology rotation. Students' perceptions and feedback should be assessed in further research on this topic.

## Conclusions

In conclusion, this study provides evidence that learning outcomes during the ophthalmology rotation were insufficient, particularly regarding more advanced ophthalmological topics such as the anatomy and physiology of the posterior segment and extraocular muscles. The significant improvement in the Red Eye and Primary Care Attention domain suggests that the current rotation model effectively teaches basic ophthalmological concepts and primary care skills. However, the decline in performance in key areas related to posterior segment diseases, such as diabetic retinopathy, indicates that the curriculum needs to be revised to provide a more comprehensive understanding of ophthalmological conditions that general practitioners will likely encounter in clinical practice.

Furthermore, the study underscores the need for a more structured and intensive approach to ophthalmology education, particularly in areas requiring practical skills. The insufficient improvement seen in this study aligns with previous reports indicating that non-ophthalmologist physicians often lack confidence in handling ophthalmic cases. This education gap could lead to increased healthcare costs and delays in managing time-sensitive conditions. Future studies should aim for a larger sample size and longer questionnaires and investigate the efficacy of alternative teaching methods.

## Appendices

Pre-Rotation Questionnaire

1. Where are photoreceptor cells located in the eye?

   - Retina

   - Cornea

   - Sclera

   - Uvea

2. Which of the following eye pathologies is associated with preauricular lymphadenopathy?

   - Viral Conjunctivitis

   - Blepharitis

   - Bacterial Conjunctivitis

   - Pterygium

3. A 65-year-old hyperopic woman presents for an ophthalmology consultation with acute unilateral visual acuity loss, pupils in mydriasis and non-reactive to light, and an intraocular pressure of 40 mmHg in the dilated eye. Which of the following pathologies has the highest pre-test probability?

   - Acute Angle-Closure Glaucoma

   - Diabetic Retinopathy

   - Retinal Detachment

   - Hypertensive Retinopathy

4. Among the following eye pathologies, which can be treated in a primary health care unit, generally not requiring referral to an ophthalmologist?

   - Glaucoma

   - Uveitis

   - Blepharitis

   - Hypertensive Retinopathy

5. The imbalance of the extraocular muscles is associated with which of the following ophthalmological pathologies?

   - Strabismus

   - Hordeolum

   - Keratoconus

   - Pterygium

6. A patient arrives at the ophthalmology office. Which of the following pathologies has the highest pre-test probability?

   - Uncorrected Refractive Error

   - Conjunctivitis

   - Diabetic Retinopathy

   - Age-Related Macular Degeneration

7. Which of the following pathologies is characterized by optic nerve damage, usually associated with increased intraocular pressure, and progressive visual field loss?

   - Glaucoma

   - Uncorrected Refractive Error

   - Dacryocystitis

   - Vitreous Hemorrhage

8. Which of the following pathologies is characterized by inflammation of the eyelid margin, associated with hyperemia of the eyelid and conjunctival margins, as well as crusting at the eyelid margin?

   - Blepharitis

   - Age-Related Macular Degeneration

   - Pigmentary Glaucoma

   - Uveitis

9. Considering the anatomy of the eyeball, which of the following structures is not part of the outer layer of the eyeball?

   - Cornea

   - Sclera

   - Choroid

   - Limbal

10. A patient arrives for a medical consultation presenting with subconjunctival hemorrhage that began in the last hour. What should be the generally indicated medical conduct?

    - Refer to an ophthalmologist

    - No need for therapeutic conduct

    - Topical NSAIDs three times a day for one week

    - Refer with a request for urgent vitrectomy

11. The visual acuity considered "normal" is:

    - 20/20

    - 20/15

    - 25/20

    - 15/20

Post-Rotation Questionnaire

1. From which of the following tests can we diagnose strabismus?

   - Cover Test

   - Visual Acuity Test

   - Tonometry

   - Pupillary Reflex Test

2. The Tropicamide eye drops is commonly used in ophthalmology practice to:

   - Dilate the pupil

   - Test corneal sensitivity

   - Measure intraocular pressure

   - Test the photomotor reflex

3. A patient arrives at the office for a consultation with an ophthalmologist presenting the following symptoms: Visual insufficiency and headache. Which of the following pathologies has the highest pre-test probability?

   - Uncorrected Refractive Error

   - Retinal Venous Occlusion

   - Normal Tension Glaucoma

   - Conjunctivitis

4. A deficiency in the drainage of aqueous humor from the anterior chamber is associated with the pathophysiology of:

   - Glaucoma

   - Retinal Detachment

   - Pterygium

   - Uncorrected Refractive Error

5. Which of the following pathologies is associated with infection of the eyelid glands?

   - Refractive Error

   - Hordeolum

   - Pterygium

   - Glaucoma

6. Which of the following pathologies is considered an ophthalmological emergency?

   - Hyphema

   - Diabetic Retinopathy

   - Blepharitis

   - Subconjunctival Hemorrhage

7. Which type of conjunctivitis is most common in clinical practice?

   - Bacterial Conjunctivitis

   - Viral Conjunctivitis

   - Allergic Conjunctivitis

   - Fungal Conjunctivitis

8. Uveitis is a common ophthalmological condition, defined as inflammation of the uveal tissue. In this context, which of the following structures is not a component of the uveal tissue?

   - Conjunctiva

   - Iris

   - Ciliary Body

   - Choroid

9. Which of the following eye pathologies has a recommendation for referral to an ophthalmologist instead of treatment at a primary care unit?

   - Acute Glaucoma

   - Subconjunctival Hemorrhage

   - Blepharitis

   - Hordeolum

10. Floaters in vision is a symptom often ignored in clinical practice. This symptom may precede management for which of the following eye pathologies?

    - Retinal Detachment

    - Keratoconus

    - Conjunctivitis

    - Glaucoma

11. The fovea, considered the point of highest visual acuity of the eye, is located in the:

    - Cornea

    - Sclera

    - Retina

    - Uvea
